# Computer-Aided Design and Computer-Aided Manufacturing (CAD/CAM) Fabricated Laminate Veneers in Aesthetic Rehabilitation: A Case Report

**DOI:** 10.7759/cureus.67556

**Published:** 2024-08-23

**Authors:** Pooja M Chitlange, Seema R Kambala, Dhanashree A Minase, Khushbu Doshi, Pragati Agarwal, Vedant Pathak

**Affiliations:** 1 Department of Prosthodontics and Crown and Bridge, Sharad Pawar Dental College, Datta Meghe Institute of Higher Education and Research, Wardha, IND

**Keywords:** lithium disilicate, cut-back technique, aesthetics, porcelain laminate veneers, cad/cam

## Abstract

Porcelain laminate veneers are a popular cosmetic dentistry treatment for correcting discoloured, worn, misaligned, gapped, chipped, or crooked teeth. The restorative material utilized in the indirect method can be processed using CAD/CAM (computer-aided design and computer-aided manufacturing) technology or conventional technique, which is highly sensitive. Due to its multiple benefits, digital technology is growing quickly and has opened up a lot of new opportunities for dental practitioners. These days, CAD/CAM is a helpful technique that enables the creation of monolithic restorations for ceramic materials, which is most recently utilized in the field of ceramic veneers as well as digital impression capturing and digital designing as part of treatment planning. This case study details the methodical process of creating laminate veneers for a patient who wants to enhance the look of their anterior teeth utilizing both traditional and CAD/CAM technology.

## Introduction

Dental ceramic veneers, sometimes referred as laminate veneers or porcelain veneers, are thin shells that are attached to the front of teeth to enhance their appearance. The correction of discoloured, worn, misaligned, gapped, chipped, or crooked teeth is a common cosmetic dental procedure. Veneers made of porcelain laminate have gained popularity as a conservative method of improving the appearance of anterior teeth. The mechanical and visual qualities of glass ceramic materials have made them a popular choice for restoring enamel loss [1, 2]. This restorative material, which is utilized in the indirect method, can be processed using CAD/CAM (Computer-Aided Design and Computer-Aided Manufacturing) technology or conventional laboratory techniques, which is very sensitive [3]. Due to its many benefits, digital technology is developing rapidly and has opened up a wide range of new opportunities for dental practice [4].

Currently, CAD/CAM is a helpful technique that enables the creation of monolithic restorations for ceramic materials and was most recently utilized in the field of ceramic veneers as well as digital impression capturing and digital design as part of treatment planning. Lithium disilicate, the substance used to create ceramic laminate veneers, is reinforced by ceramic and can be manufactured using press techniques or CAD/CAM systems. For CAD/CAM, this material is supplied as recrystallized blocks of lithium metasilicate with cores of both disilicate and metasilicate, bringing its flexural strength down to 130 ± 30 MPa. This makes it possible for the restoration's shape to be obtained during the milling process [5].

After that, the ceramic is recrystallized for 20 to 25 minutes at 850°C. Lithium disilicate crystallizes and lithium metasilicate dissolves throughout this process, giving the restoration its ultimate translucency. Ultimately, both the volume percentage of crystals and the flexural strength rise to 70% and 360-400 MPa, respectively. The definitive restoration might not have the best teeth optical qualities at this time [6]. Three techniques can be used to characterize the incisal third in this situation: (a) cut-back technique, (b) staining technique, and (c) layering approach [7, 8]. This case study details the methodical process of creating laminate veneers for a patient who wants to enhance the look of their anterior teeth utilizing both traditional and CAD/CAM technology.

## Case presentation

**Figure 1:**
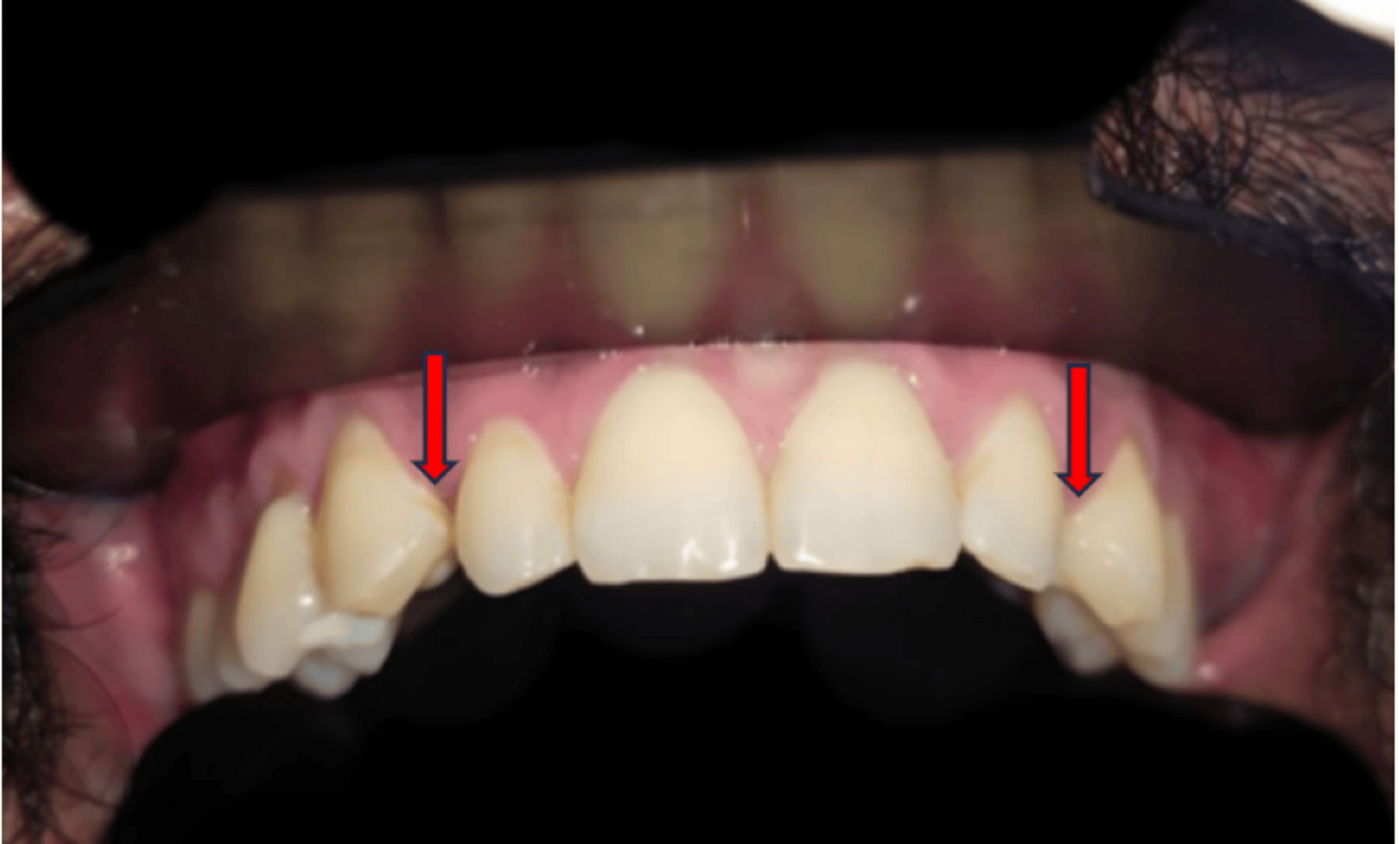
Spacing between the lateral incisor and canine The red arrow shows the spacing between the teeth

A 29-year-old male patient visited the Department of Prosthodontics and Crown and Bridge with concerns regarding the appearance of his maxillary anterior teeth, notably the presence of space between the lateral incisor and canine of both the quadrants, as depicted in Figure 1. The patient was informed about various treatment options during the clinical evaluation, including laminate veneers, orthodontic treatment, and composite restorations. The patient preferred a conservative approach to improve the looks of his smile over orthodontic treatment because of it is time-consuming nature.

Treatment planning

**Figure 2:**
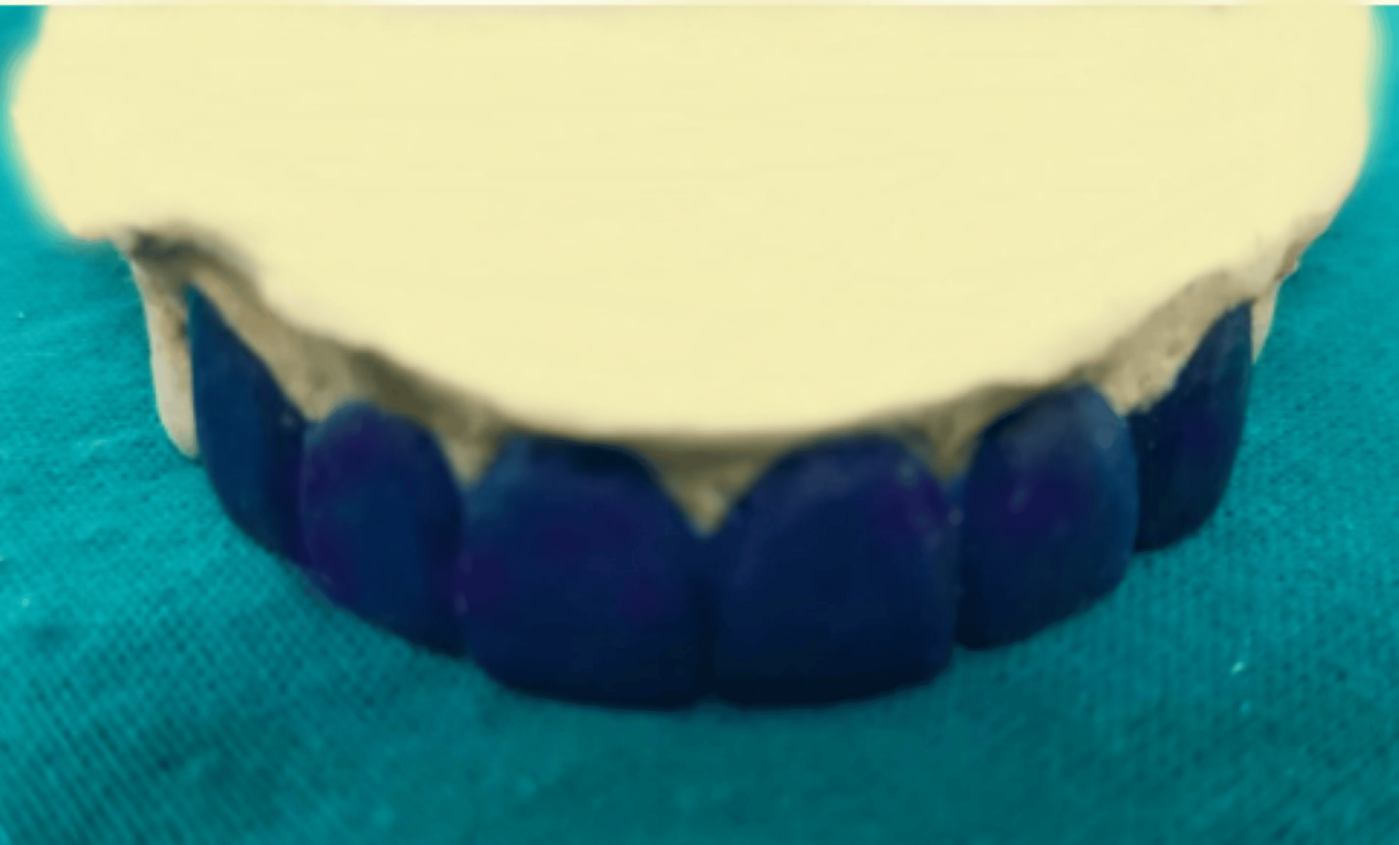
Diagnostic wax-up

After a thorough clinical assessment, porcelain laminate was found to be the best treatment option for the patient. Six maxillary anterior laminate veneers were created utilizing the combination of conventional impression technique and CAD/CAM process as part of the treatment plan. Before beginning the procedure, the patient was fully informed about the course of treatment and informed consent was obtained. Following comprehensive clinical and radiographic evaluations, it was established that the patient exhibited a low risk of dental caries with no active lesions or signs of periodontal disease. A diagnostic impression was made to assess the spacing, and a diagnostic wax-up was performed. Tooth preparations for teeth 13-23 on the diagnostic cast involved reducing 0.5 mm of tooth structure from both the labial and incisal surfaces. The diagnostic wax-up was executed using type 2 inlay wax, as depicted in Figure 2. The diagnostic wax-up was presented to the patient and he agreed to proceed with the treatment.

Tooth preparation and impression making

**Figure 3:**
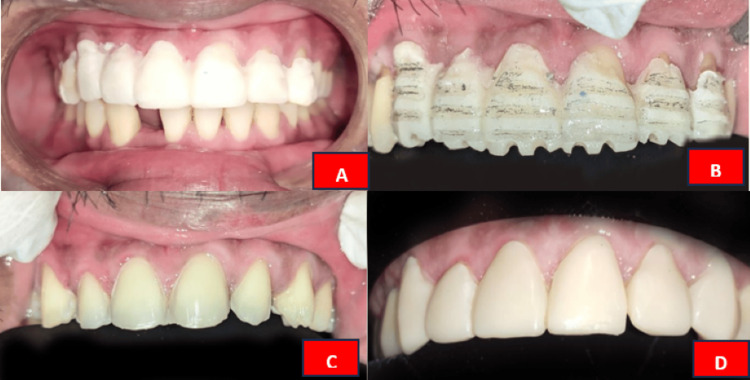
A: Fabrication of temporary prosthesis before tooth preparation; B: Orientation grooves; C: Tooth preparation for laminate prosthesis; C: Fabrication of temporary prosthesis after tooth preparation.

The pre-evaluation temporization technique was used to fabricate the temporary restoration of 0.5mm using Luxatemp Ultra (DMG, Ridgefield Park, NJ) and cemented with the help of temporary luting cement as shown in Figure 3A. The teeth were prepared for laminate veneers with a focus on minimal tooth reduction. The orientation grooves were made for the vestibular reduction using 834-016 diamond bur (0.3 mm depth) as shown in Figure 3B. A 1 mm reduction from the labial surface was performed with temporary restoration using 850-016 diamond bur without compromising the contact points between the central incisors. A chamfer finish line was placed at the subgingival margin using 850-014 diamond bur, and the incisal edge was reduced by approximately 1 mm, as shown in Figure 3C. Following preparation, the teeth were cleaned, and a retraction cord was placed to expose the finish lines. An impression of the prepared teeth was then made using a two-stage impression technique (firstly, the cellophane sheet was adapted and the putty impression was made; cellophane sheet was removed, putty impression was scraped off and second impression was made using light body elastomeric impression material). The impression was made under uniform pressure in the mouth. Subsequently, the impression was poured with type IV gypsum product. The A2 shade was chosen using the VITA Toothguide 3D-MASTER (VITA, Yorba Linda, CA) under natural daylight conditions. Temporary restoration was given using Luxatemp Ultra as decipated in Figure 3D.

CAD design and milling

**Figure 4:**
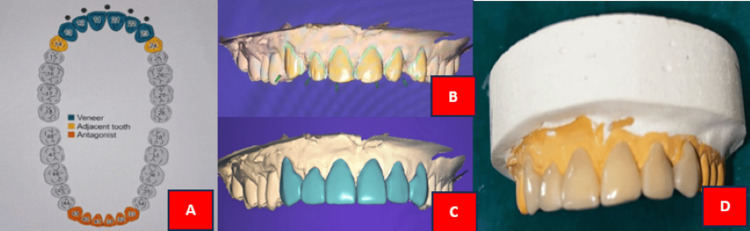
A: Teeth selection in DentalCAD software; B: Path of insertion; C: Designed final prosthesis; D: Fabricated final laminate prosthesis Image credit: Pooja Chitlange

The cast was scanned with a laboratory scanner (inExo X5; Dentsply Sirona, Charlotte, NC) to generate a 3D model of the prepared teeth in DentalCAD software (exocad, Darmstadt, Germany). The veneer design was tailored to meet the patient's aesthetic preferences and the natural shape of their teeth. A cut-back technique was used to craft the incisal edge, enhancing both characterization and translucency. After finalizing the design, the veneers were digitally placed on the 3D model and the milling procedure was started. Using a CAD/CAM milling machine (inLab MC X5; Dentsply Sirona), the veneers were milled from lithium disilicate ceramic material (CEREC Tessera^TM^; Dentsply Sirona) sourced from an A2 shade block (Figure 4).

Try-in and cementation

**Figure 5:**
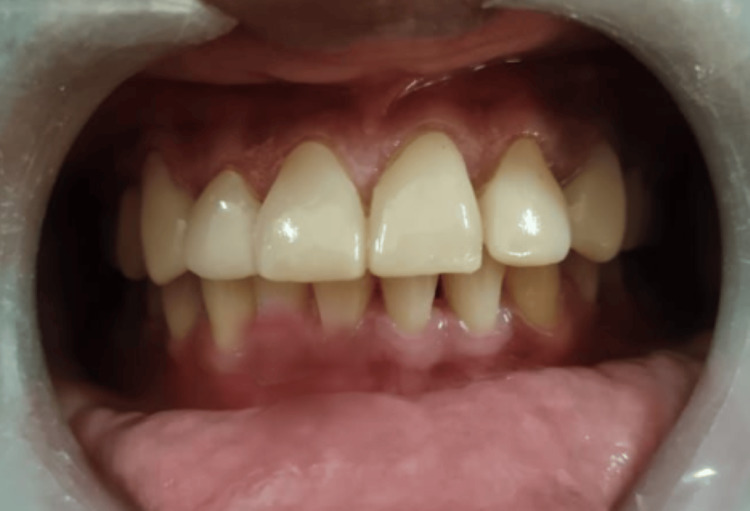
Cementation of the final laminate prosthesis

The milled veneers were placed in the patient's mouth for a try-in to assess fit, occlusion, and aesthetic appearance. In preparation for bonding, the inner surfaces of the veneers were treated: they were etched using 30% hydrofluoric acid, rinsed thoroughly, and then treated with a silane coupling agent. Meanwhile, the prepared teeth were isolated and treated with 37% orthophosphoric acid, followed by an application of a dentine bonding agent as per the manufacturer's guidelines. A self-cure cement, luting resin was utilized for cementation. The veneers were meticulously positioned onto the prepared teeth after the cement was applied on their intaglio surface (Figure 5). To get an exact fit and finish, extra cement was scraped off. To ensure the durability of the restorations, the patient was given advice on oral hygiene and recommended to maintain good oral health practices. The follow-up was conducted at one-week, one-month, and three-month interval.

## Discussion

In the recent years, restorative and prosthetic dentistry has been transformed drastically by the use of CAD/CAM technology in prosthesis manufacturing, especially in the production of laminate veneers. Patients can have veneers designed precisely and efficiently in a single visit using this technique. This also saves a lot of chair-side time and decreases laboratory procedures [8]. The impression is the first step to start with the fabrication of laminate veneers, which can be made using conventional technology or digital technology (intraoral scanner). The cast created by the conventional impression technique can be converted into digital information using a laboratory scanner. Using specialist CAD software, this digital data is then used to design and create the veneer, enabling customization to match the patient's natural tooth morphology and colour [9].

The CAM component takes over after the design is complete: using a milling machine to create the veneer from a ceramic block, especially with lithium disilicate that is prized for both its strength and aesthetics. Layering techniques can be best employed for the characterization of this material in order to improve its optical qualities and guarantee a natural look. Excellent marginal fit and finish of the final veneers produce high patient satisfaction [5].

Clinical research has shown that veneers fabricated with CAD/CAM technology not only yield better aesthetic results but also have encouraging long-term success rates. As an illustration of their dependability as a therapeutic choice, a retrospective study revealed chairside CAD/CAM ceramic laminate veneers to have favourable five-year survival rates [10]. A recent development in restorative dentistry is the incorporation of CAD/CAM technology in the porcelain laminate fabrication process. Considering all the aspects, the use of CAD/CAM technology in the fabrication of laminate veneers is a noteworthy development for dental practitioners, providing a restorative dentistry experience that combines accuracy, efficiency, and superior aesthetics [6].

The capabilities of CAD/CAM systems are anticipated to grow as technology progresses, greatly improving the science and art of dental restoration. In this case report, the aesthetic restoration of anterior teeth is done using a combination of conventional technique and digital technology. The impression for laminate veneer was created by the conventional impression technique and fabrication and milling were done using CAD/CAM technology.

## Conclusions

Laminate veneers can now be made more precisely and predictably by using CAD/CAM technology, simplifying the treatment process. This case study shows how CAD/CAM may be successfully used to fabricate porcelain laminate veneers, giving the patient a more pleasing aesthetic result.
